# Common angiotensin receptor blockers may directly modulate the immune system via VDR, PPAR and CCR2b

**DOI:** 10.1186/1742-4682-3-1

**Published:** 2006-01-10

**Authors:** Trevor G Marshall, Robert E Lee, Frances E Marshall

**Affiliations:** 1 Autoimmunity Research Foundation, Thousand Oaks, California 91360, USA; 2 Black Hawk College, Moline, Illinois 61443, USA; 3 Los Robles Regional Medical Centre, Thousand Oaks, California 91360, USA

## Abstract

**Background:**

There have been indications that common Angiotensin Receptor Blockers (ARBs) may be exerting anti-inflammatory actions by directly modulating the immune system. We decided to use molecular modelling to rapidly assess which of the potential targets might justify the expense of detailed laboratory validation. We first studied the VDR nuclear receptor, which is activated by the secosteroid hormone 1,25-dihydroxyvitamin-D. This receptor mediates the expression of regulators as ubiquitous as GnRH (Gonadatrophin hormone releasing hormone) and the Parathyroid Hormone (PTH). Additionally we examined Peroxisome Proliferator-Activated Receptor Gamma (PPARgamma), which affects the function of phagocytic cells, and the C-CChemokine Receptor, type 2b, (CCR2b), which recruits monocytes to the site of inflammatory immune challenge.

**Results:**

Telmisartan was predicted to strongly antagonize (Ki≈0.04nmol) the VDR. The ARBs Olmesartan, Irbesartan and Valsartan (Ki≈10 nmol) are likely to be useful VDR antagonists at typical in-vivo concentrations. Candesartan (Ki≈30 nmol) and Losartan (Ki≈70 nmol) may also usefully inhibit the VDR. Telmisartan is a strong modulator of PPARgamma (Ki≈0.3 nmol), while Losartan (Ki≈3 nmol), Irbesartan (Ki≈6 nmol), Olmesartan and Valsartan (Ki≈12 nmol) also seem likely to have significant PPAR modulatory activity. Olmesartan andIrbesartan (Ki≈9 nmol) additionally act as antagonists of a theoretical modelof CCR2b. Initial validation of this CCR2b model was performed, and a proposed model for the AngiotensinII Type1 receptor (AT2R1) has been presented.

**Conclusion:**

Molecular modeling has proven valuable to generate testable hypotheses concerning receptor/ligand binding and is an important tool in drug design. ARBs were designed to act as antagonists for AT2R1, and it was not surprising to discover their affinity for the structurally similar CCR2b. However, this study also found evidence that ARBs modulate the activation of two key nuclear receptors-VDR and PPARgamma. If our simulations are confirmed by experiment, it is possible that ARBs may become useful as potent anti-inflammatory agents, in addition to their current indication as cardiovascular drugs.

## Background

### Why would ARBs have dose-dependent efficacy?

Angiotensin Receptor Blockers (ARBs) act as antagonists of the AngiotensinII Type1 receptor (AT2R1) [Swiss-Prot:P30556], and were designed to treat moderate hypertension. Although ARBs have been marketed for nearly a decade, their mode of action is not fully understood, and debate still rages whether Angiotensin Converting Enzyme Inhibitors (ACEI) or ARBs are superior at reducing ultimate mortality due to cardiovascular dysfunction.

An editorial in the New England Journal of Medicine concluded [1]:

"in two recently reported clinical trials in which the investigators were allowed to increase the dose of Losartan gradually to 100 mg per day, there was a significant reduction in the incidence of heart failure among high-risk patients; this finding raises the important question of whether higher doses of Losartan might have been more effective in reducing the rates of cardiovascular events"

Yet in-vitro studies [2] have shown that the ARBs produce an efficient and total blockade of the Angiotensin II Type 1 receptor (AT2R1) at doses much lower than this editorial was contemplating. There should be no dose related effects once a total receptor blockade is place, so the obvious question arises "how can an ARB have dose-dependent efficacy?"

It is accepted that diabetic nephropathy is beneficially affected by ARBs [3-6], yet again the mechanisms, and optimal dosage, remain elusive. A study using Irbesartan noted dosage-dependant efficacy, with significantly greater protection at 300 mg/day versus 150 mg/day [4].

Schieffer, et.al. [7], found that ARBs appeared to exert stronger systemic anti-inflammatory and anti-aggregatory effects compared with ACEIs in Atherosclerosis. Luno, et.al. [8], recently reviewed studies which have shown that ACE Inhibitors (ACEI) did not always lead to the same clinical outcome as ARBs, especially where the patient was suffering from inflammatory diseases such as diabetes.

The reason for this is not immediately obvious, as ACE's function is to cleave the octapeptide Angiotensin II from Angiotensin I. The AngiotensinII then binds to AT2R1 receptors on the activated phagocytes, an action inhibited by the ARBs. Interrupting either pathway, with either ACEI or ARBs, should have the same effect – the activated phagocytes will be denied Angiotensin II bound at their receptors.

Waterhouse, et.al. [9], and Marshall, et.al. [10], noted that patients with autoimmune disease were anecdotally reporting that ARBs prescribed for hypertension caused a noticeable change in their perceived immune disease symptoms, a change not easily explained in terms of hypertension, or hypotension, alone. We consequently decided to investigate whether molecular modelling could help define precise mechanism(s) of action of the ARBs upon inflammatory disease. Do they perhaps act as antagonists for receptors other than AT2R1? Immune system receptors, for example?

### Identifying target nuclear and transmembrane receptors

#### 1. The VDR

The T-helper Type 1 (Th1) immune response is usually defined as one which generates significant quantities of the cytokine Interferon-gamma [11]. Many chronic diseases are associated with Th1 inflammation [12], including atherosclerosis [13], diabetes [14], and perhaps even asthma [15].

Generation of Interferon-gamma in a Th1 activated macrophage catalyzes its mitochondrial production of the secosteroid hormone 1,25-dihydroxyvitamin-D (1,25-D) by as much as 30-fold [16]. 1,25-D is the active secosteroid of the Vitamin-D metabolism [9]. This steroid's presence is often ignored by clinical medicine, since it circulates in low concentrations (typically 75 picomoles/Litre, 29 pg/ml), which are very difficult to measure. Yet 1,25-D and its receptor, the Vitamin-D Receptor (VDR) [Swiss-Prot:P11473], are expressed in over 30 target tissues, and their expression is tightly coupled with regulators as ubiquitous as GnRH (Gonadatrophin hormone releasing hormone) [17], and the Parathyroid Hormone(PTH) [18].

Ripple-down effects of VDR activation include changes not only to the androgens and thyroid hormones, but also to ACTH, Insulin Receptors, P450C1, and many other biologically important metabolites [18,46].

In patients with severe Th1 immune disease, clinical observations [9,10] indicated that the administration of the ARB Olmesartan, at a concentration in excess of that needed for full AT2R1 antagonism, often causes the level of circulating 1,25-D to drop.

We therefore decided to target the VDR nuclear receptor [19] for further study.

#### 2. Peroxisome Proliferator Activated Receptors (PPARs)

Benson, et.al. reported [20] that the ARB 'Telmisartan' seems to act both as an agonist and antagonist of Peroxisome Proliferator Activated Receptor gamma (PPARgamma) [Swiss-Prot:P37231], a nuclear hormone receptor from the same 'NR1' subfamily as VDR. The PPARs act as anti-inflammatory transcription factors [21]. Part of this anti-inflammatory regulation is mediated through negative interference between PPARs and nuclear factors such as NF-kappaB. Ligands of PPAR may affect the inflammatory response in diseases as wide-ranging as Inflammatory Bowel Diseases, Atherosclerosis, Parkinson's Disease and Alzheimer's [22]. Clearly, it is important to know exactly how the ARBs might affect PPARgamma.

#### 3. C-C chemokine receptor type 2 (CCR2b)

Monocyte chemotactic protein-1 (MCP-1) binding to its receptor, CCR2b [EMBL:BC095540], plays an important role in a variety of diseases involving infection, inflammation, and/or injury [23,24]. CCR2b recruits monocytes to the sites of tissue damage. The monocytes later differentiate to macrophages and/or polymorphonucleated 'giant' cells.

CCR2b belongs to the same family of 7-Transmembrane G-Protein Coupled Receptors (GPCRs) [25] as does AT2R1, and the similarities between these two GPCRs, together with the clinical observations [9,10], supported the addition of CCR2b to this study.

## Results

### Validation of 'AutoDock' simulation software

It was decided to use automated docking of the ligands so as to minimize subjective factors which might arise if the ligands were fitted into the binding pockets manually. The Scripps' package, AutoDock [26-28], was selected for this task. Toprakci, et.al. [29], recently compared the Ki values estimated by AutoDock for ten inhibitors of human monoamine oxidase-B, with the values of Ki which had been determined by experiment. In every case, there was less than one order of magnitude difference between the experimentally determined Ki, and the value estimated by computer simulation of the ligand-bound enzyme. Chen, et.al. [30], also concluded that AutoDock provided accurate estimation of ligand-DNA binding parameters.

We were able to compare calculated Ki for some of our docking experiments with published values, and similarly found excellent agreement. For example, we validated our PPARgamma model by docking the ligand GI262570 (Farglitazar), essentially as predicted by the data of Xu, et.al. [31].

It is important to understand that the 'Lamarckian genetic algorithm' used by AutoDock does not guarantee convergence to an optimal solution. The existence of the 'optimal' solution, amongst any set of docking results, only becomes assured as the number of docking attempts tends to infinity. Considerable computing power was expended in order to maximize the likelihood that this study identified the lowest energy docking configurations. Additionally, the algorithm's convergence parameters were manually adjusted whenever successive docking runs were not returning consistent minima.

### ARBs exhibit a strong affinity for VDR ligand binding pocket

In order to maximize reliability, two discrete models were used for the ligand binding pocket of the VDR, extracted from two separate X-ray generated structures. The first model was "The crystal structure of the nuclear receptor for vitamin D bound to its natural ligand" [32] [PDB:1DB1], while the second was the VDR bound to the agonist TX522 [33] [PDB:v].

There was no significant difference between the results obtained from either VDR structure. Table 1 shows the predicted inhibition constants (Ki), in nanomoles, for each of the ARBs binding into [PDB:1DB1] and [PDB:1TXI].

**Table 1:** Estimated Inhibition Constant, Ki (nmol), for ARBs docking into several immune system receptors.

	Olmesartan	Telmisartan	Valsartan	Irbesartan	Candesartan	Losartan
VDR,1DB1	12, 27	0.038	14	10	35	77
VDR,1TXI	10,34	0.039	14	12	30	74
PPAR	12	0.29	12	6	61	3
CCR2b *	9*	25*	22*	9*	39*	25*
AT2R1 *	0.10*	0.10*	0.3*	0.17*	1.5*	0.50*

*Note 1: CCR2b and AT2R1 are theoretical models, and may not be reliable (see text)

Note 2: (conventional ligand binding data): 1,25-dihydroxyvitamin-D docks into VDR (PDB:1DB1) with Ki = 0.029 nmol and into VDR (PDB:1TXI) with Ki= 0.059 nmol

TX522 docks into VDR (PDB:1DB1) with Ki = 0.071 nmol and VDR (PDB:1TXI) with Ki = 0.12 nmol

TAK779 docks into putative CCR2b with Ki = 10 nmol

GI262570 docks into PPAR (PDB:1FM9) with Ki = 0.040 nmol.

**Figure 1:**
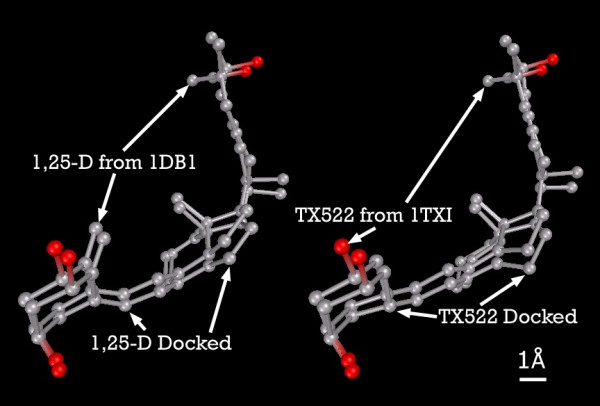
**1,25-D and TX522 with superimposed X-ray and VDR-docked configurations**. Note: Carbon atoms shown as grey, oxygen as red. Hydrogens not displayed.

As a further check of model validity, 1,25-D was initially docked into [PDB:1DB1] with a Ki = 0.03 nmol and into [PDB:1TXI] with Ki = 0.06 nmol. TX522 was then docked into [PDB:1DB1] with Ki = 0.07 nmol and [PDB:1TXI] with Ki = 0.12 nmol. The difference between the crystal structure of the ligands and the predicted docked conformations was very small (Figure 1), and seems primarily due to AutoDock's reliance upon grid-based energy calculations.

**Figure 2:**
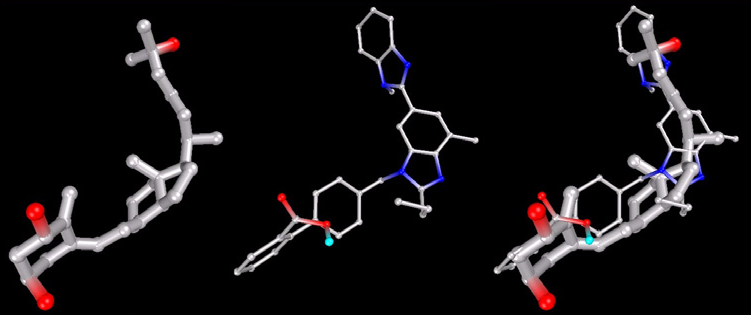
**VDR-docked configurations for 1,25-D and Telmisartan, separately and superimposed**. Note: Models depicted as "thick" and "thin" solely for visual clarity. Carbon atoms shown as grey, oxygen as red, nitrogen shown as blue, polar hydrogen as blue-white. Non-polar hydrogens not displayed.

The ARB 'Telmisartan' had a strong affinity for the VDR, with Ki≈0.04 nmol into either structure. This value is close to that achieved by 1,25-D itself, which yielded Ki≈0.03 nmol into [PDB:1DB1] and Ki≈0.09 nmol into [PDB:1TXI]. Telmisartan docked with a conformation uncannily similar to 1,25-D (see Figure 2).

Irbesartan and Valsartan gave predicted Ki values in the 10–14 nanomolar region, probably indicating significant antagonistic action at concentrations safely achievable in-vivo.

Olmesartan similarly predicted useful Ki values, ranging from 10 to 34 nmol. Particularly interesting is that two distinct conformations were identified.

**Figure 3:**
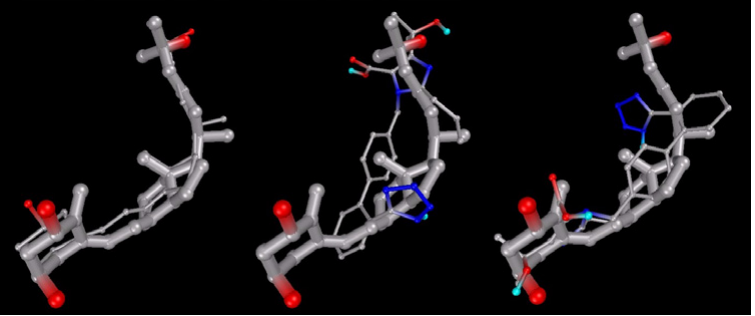
**VDR-docked configurations for 1,25-D and Olmesartan, with superimposition showing both conformations**. Note: Models depicted as "thick" and "thin" solely for visual clarity. Carbon atoms shown as grey, oxygen shown as red, nitrogen as blue, polar hydrogen as blue-white. Non-polar hydrogens not displayed.

Figure 3 shows that Olmesartan docked in each conformation, one with its imidazole terminus near the triol of 1,25-D. The second focused on the seco terminus of 1,25-D.

Losartan docked with a Ki around 70 nanomolar, Candesartan around 30 nanomolar. These are likely also significant antagonists, but higher dosage levels would be necessary.

### Hydrogen bonds and hydrophobic contacts during docking with the VDR

**Figure 4:**
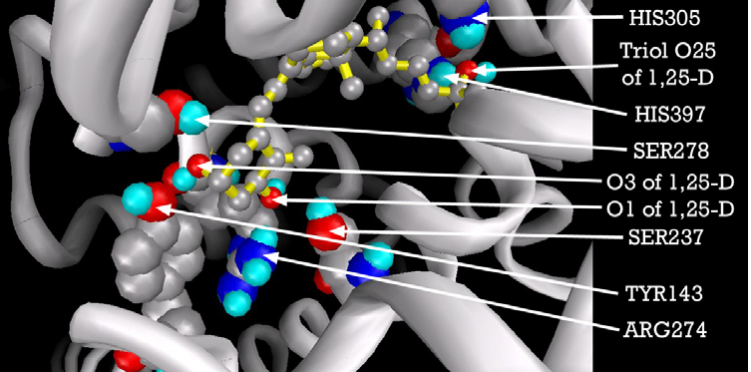
**VDR binding pocket showing primary 1,25-D docking residues**. Note: 1,25-D depicted with yellow backbone for visual clarity. Carbon atoms shown as grey, oxygen as red, nitrogen as blue, polar hydrogen as blue-white. Non-polar hydrogens not displayed. Residues displayed as 'CPK' charge spheres, ligand in 'ball and stick' format.

Figure 4 shows the ligand binding pocket of the VDR with 1,25-D docked into it, highlighting those residues with which 1,25-D forms hydrogen-bonds.

**Figure 5:**
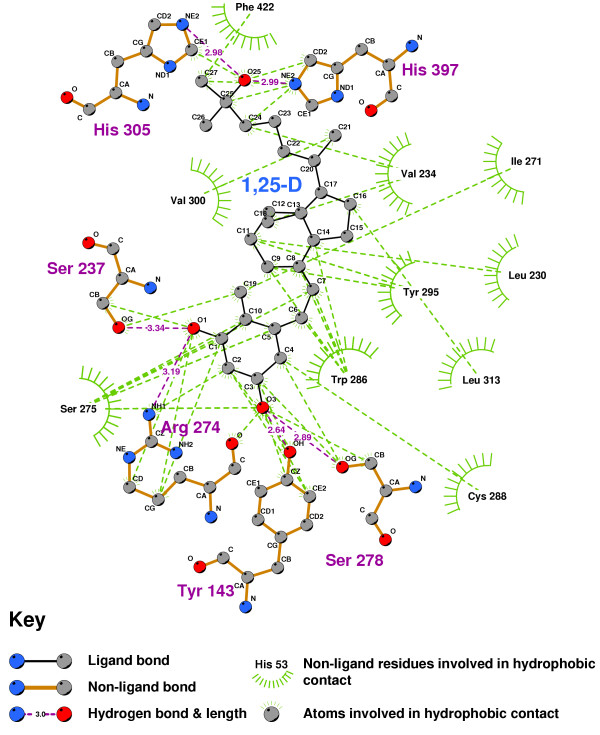
**2D LigPlot of 1,25-D bound into the VDR ligand binding pocket**. Note: The core structure of the hydrogen-bonded residues is expanded to a 'ball-and-stick' format, so as to show the atoms involved in hydrogen bond formation.

Figure 5 is a 2D representation of the 3D structure of Figure 4, created with Ligplot [53,54]. The hydrogen bonds were identified with HBPLUS [55,56], as were the hydrophobic contacts formed between 1,25-D and the VDR residues. The core structure of the hydrogen-bonded residues is expanded to a 'ball-and-stick' format so as to show which atoms are involved in hydrogen bond formation.

A double hydrogen bond was formed from the oxygen of the triol group of 1,25-D, both to the imidazole nitrogen of HIS305, and to the imidazole nitrogen of HIS397. Another hydrogen bond extends from the 1-hydroxyl oxygen to the aminoacetal of ARG274 and the hydroxyl of SER237, and another pair from the ligand's O3 oxygen to SER278 and TYR143.

**Figure 6:**
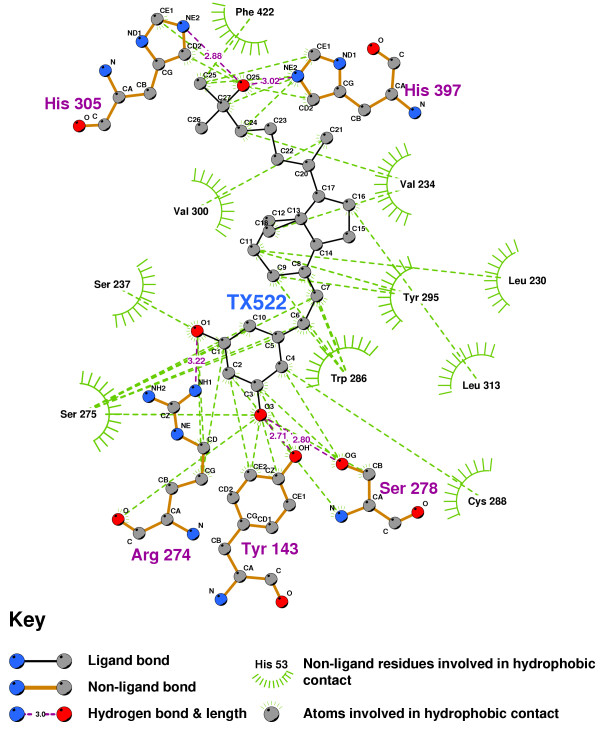
**The VDR agonist TX522 in the VDR ligand binding pocket**. Note: The core structure of the hydrogen-bonded residues is expanded to a 'ball-and-stick' format, so as to show the atoms involved in hydrogen bond formation.

Figure 6 shows that the VDR agonist TX522 [42] also forms a double hydrogen bond between the oxygen of its triol group, the imidazole of HIS397, and the imidazole of HIS305. The 3-hydroxyl-oxygen is hydrogen-bonded to TYR 143 and SER278, while the 1-hydroxyl-oxygen forms a hydrogen bond with the aminoacetal of ARG274. No hydrogen bond is formed with SER237, presumably due to a lowered affinity consequent upon the removal of the C19 position carbon from 1,25-D(cf.Figure 4).

**Figure 7:**
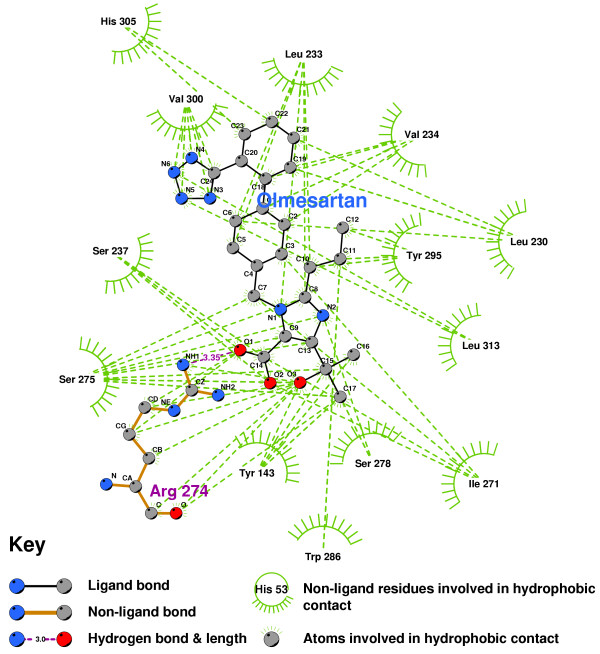
**Olmesartan bound into the sterol terminus of the VDR binding pocket**. Note: This is the 12 nanomolar conformation of Olmesartan in the binding pocket. The core structure of the hydrogen-bonded residues is expanded to a 'ball-and-stick' format, so as to show the atoms involved in hydrogen bond formation.

The Ki = 12E-9 configuration of Olmesartan (Figure 7), forms a hydrogen bond from its imidazole terminal hydroxyl to ARG274. Olmesartan forms only hydrophobic contacts with the key VDR binding residues TYR143, SER237, SER278 and HIS305. TYR143 is especially important. It is part of the 'hinge region,' and key for VDR transcriptional activity [51,57]. It is thus almost certain that Olmesartan will function as a VDR antagonist.

**Figure 8:**
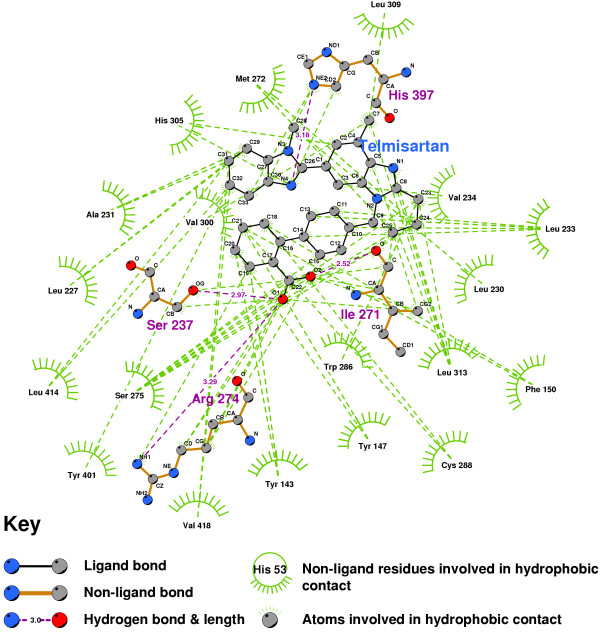
**Telmisartan docked into the VDR ligand binding pocket**. Note: Telmisartan is a strong antagonist of the VDR's activation.

Telmisartan docks with a Ki of 0.04 nmol, so that typical in-vivo concentrations of the ARB should be sufficient to displace 1,25-D from the ligand binding domain. Figure 8 shows that that hydrogen bonds are formed to SER237, ARG274, HIS397 and ILE271, but not to TYR143. SER278 or HIS305. Telmisartan would thus seem likely to act as a very strong antagonist of the VDR, with an affinity significantly stronger than the other ARBs.

**Figure 9:**
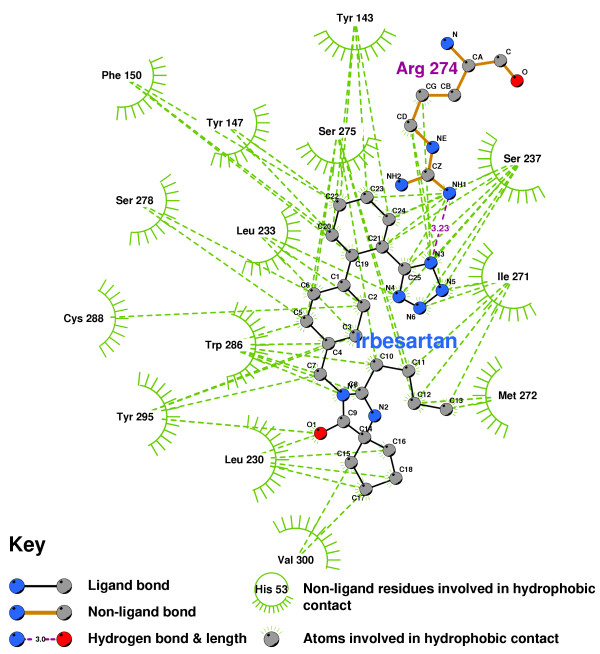
**Irbesartan docked into the VDR ligand binding pocket**. Note: The core structure of the hydrogen-bonded residues is expanded to a 'ball-and-stick' format, so as to show the atoms involved in hydrogen bond formation.

Irbesartan (Figure 9) formed a hydrogen bond between its tetrazole group and the amino of ARG274. The lack of hydrogen bonds to TYR143 and SER278 indicate that Irbesartan will be a VDR antagonist.

**Figure 10:**
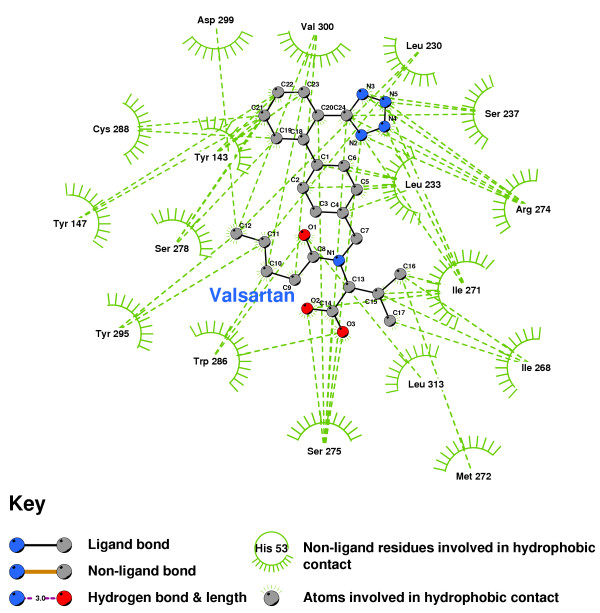
Valsartan docked into the VDR ligand binding pocket.

Valsartan, although it exhibits a potentially useful affinity as a VDR antagonist, failed to form hydrogen bonds with any key residue (Figure 10).

**Figure 11:**
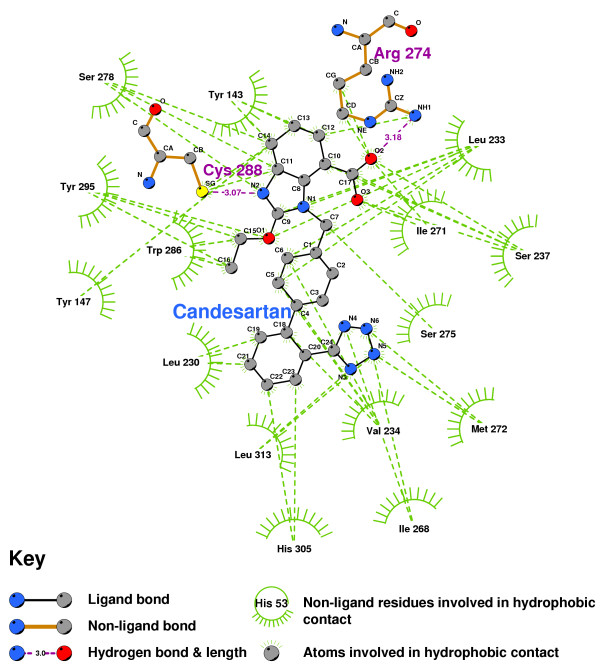
**Candesartan docked into the VDR ligand binding pocket**. Note: The core structure of the hydrogen-bonded residues is expanded to a 'ball-and-stick' format, so as to show the atoms involved in hydrogen bond formation.

**Figure 12:**
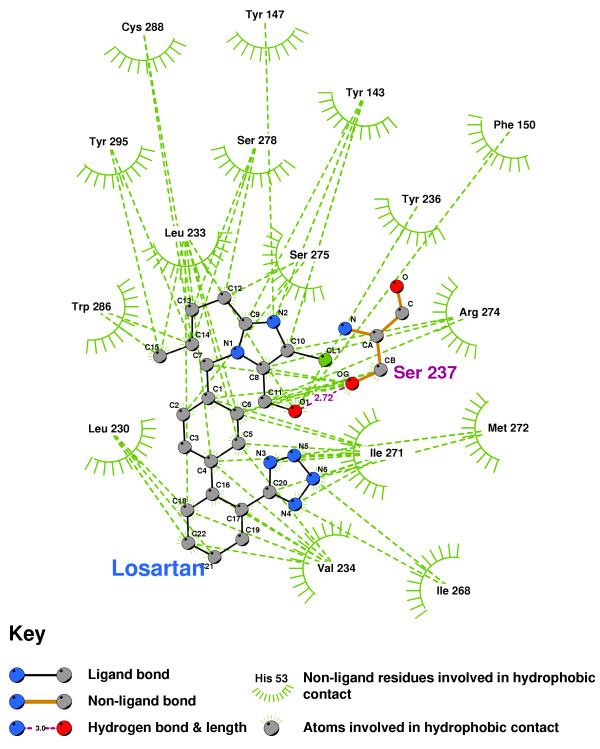
**Losartan docked into the VDR ligand binding pocket**. Note: The core structure of the hydrogen-bonded residues is expanded to a 'ball-and-stick' format, so as to show the atoms involved in hydrogen bond formation.

The imidazole of Candesartan formed a bond with the sulphur of CYS288 (Figure 11), and the imidazole terminus oxygen of Losartan hydrogen-bonded with SER237(Figure 12). Both are indicative of actions antagonistic to VDR activation.

### ARBs exhibit an affinity for PPARgamma

We extracted the coordinate data for PPARgamma from [PDB:1FM9], an X-ray structure. As model validation, the PPARgamma agonist GI262570 (Farglitazar) was docked with Ki≈0.04 nmol, close to the (approx.) 0.01 nmol predicted by the inhibition curve in figure 1A of Xu, et.al. [31].

Table 1 shows that the ARBs exhibited a strong affinity for the ligand binding pocket of PPARgamma, with Ki ranging from 0.29 to 61 nanomoles.

Telmisartan is the strongest modulator of PPARgamma (Ki≈0.3 nmol), while Losartan (Ki≈3 nmol), Olmesartan (Ki≈12 nmol), Irbesartan (Ki≈6 nmol) and Valsartan (Ki≈12 nmol) also seem likely to have significant PPAR modulatory activity. Candesartan (Ki≈ 61 nmol) may also have useful activity at a higher dosage.

### ARBs exhibit a strong affinity for CCR2b

The ARBs are designed as antagonists for the Angiotensin II Type 1 Receptor (AT2R1). This is a GPCR [36] of the "Class A (Rhodopsin-like) 7-transmembrane receptors." CCR2b is another Class A GPCR, with surprising similarity to AT2R1.

Table 2 shows the multiple sequence alignment between AT2R1 and Bovine Rhodopsin [PDB:1L9H], the prototype structure for Class A GPCRs. Table 3 shows an alignment for CCR2b vs. Rhodopsin, while Table 4 compares AT2R1 and CCR2b. It is interesting to note that CCR2b and AT2R1 both exhibit only 17% homology with Bovine Rhodopsin, while the score between them is much higher, at 27%.

**Table 2:** Multiple sequence alignment for AT2R1 and Bovine Rhodopsin (PDB:1L9H)

sp|P30556|AGTR1_HUMAN gi|21465997|pdb|1L9H|A	
sp|P30556|AGTR1_HUMAN gi|21465997|pdb|1L9H|A	
sp|P30556|AGTR1_HUMAN gi|21465997|pdb|1L9H|A	
sp|P30556|AGTR1_HUMAN gi|21465997|pdb|1L9H|A	
sp|P30556|AGTR1_HUMAN gi|21465997|pdb|1L9H|A	
sp|P30556|AGTR1_HUMAN gi|21465997|pdb|1L9H|A	
sp|P30556|AGTR1_HUMAN gi|21465997|pdb|1L9H|A	
sp|P30556|AGTR1_HUMAN gi|21465997|pdb|1L9H|A	
SeqA Name	
1 sp|P30556|AGTR1_HUMAN	

**Table 3:** Multiple sequence alignment for CCR2b and Bovine Rhodopsin (PDB:1L9H)

1kp1_A (CCR2b) gi|21465997|pdb|1L9H|A	
1kp1_A (CCR2b) gi|21465997|pdb|1L9H|A	
1kp1_A (CCR2b) gi|21465997|pdb|1L9H|A	
1kp1_A (CCR2b) gi|21465997|pdb|1L9H|A	
1kp1_A (CCR2b) gi|21465997|pdb|1L9H|A	
1kp1_A (CCR2b) gi|21465997|pdb|1L9H|A	
1kp1_A (CCR2b) gi|21465997|pdb|1L9H|A	
1kp1_A (CCR2b) gi|21465997|pdb|1L9H|A	
SeqA Name	
2 gi|21465997|pdb|1L9H|A	

**Table 4:** Multiple sequence alignment for AT2R1 and CCR2b

gi|4757938|ref|NP_000639.1|CCR2b gi|231519|sp|P30556|AGTR1_HUMA	
gi|4757938|ref|NP_000639.1|CCR2b gi|231519|sp|P30556|AGTR1_HUMA	
gi|4757938|ref|NP_000639.1|CCR2b gi|231519|sp|P30556|AGTR1_HUMA	
gi|4757938|ref|NP_000639.1| gi|231519|sp|P30556|AGTR1_HUMA	
gi|4757938|ref|NP_000639.1|CCR2b gi|231519|sp|P30556|AGTR1_HUMA	
gi|4757938|ref|NP_000639.1|CCR2b gi|231519|sp|P30556|AGTR1_HUMA	
gi|4757938|ref|NP_000639.1|CCR2b gi|231519|sp|P30556|AGTR1_HUMA	
gi|4757938|ref|NP_000639.1|CCR2b gi|231519|sp|P30556|AGTR1_HUMA	
SeqB Name	
1 gi|4757938|ref|NP_000639.1| 360	

There are no complete X-ray or NMR structures of *Homo sapiens' *Class A GPCRs in PDB, or any other public database. However, Shi, et.al. [37] had derived a theoretical model, [PDB:1KP1], which provided a basis for us to study. We tried to improve upon [PDB:1KP1] by using, inter alia, Truncated Newton energy minimization with Ponder's TINKER Tools [38,39] and homology modelling with Sali's 'Modeller' [40,41]. However, even extensive homology modelling against the Bovine Rhodopsin X-ray structure [PDB:1L9H], and other theoretical models, such as [PDB:1KPX], failed to improve upon [PDB:1KP1].

We accepted that [PDB:1KP1] was probably a valid model for CCR2b based on the detailed nature of Shi, et.al's studies [37], our failed attempts to improve upon it, and the manner in which it docked, exactly as predicted, with the CCR2b antagonist, TAK779.

A binding pocket exists between helices seven and one of [PDB:1KP1], extending back to extracellular regions one and three. Baba, et.al. [42] had measured the inhibitory effects of Tak779 on CCR2b in their laboratory, showing an experimental Ki≈9 nmol. When we docked TAK779 into our putative binding pocket, it predicted a Ki≈10 nmol, essentially identical with this experimental value.

**Figure 13:**
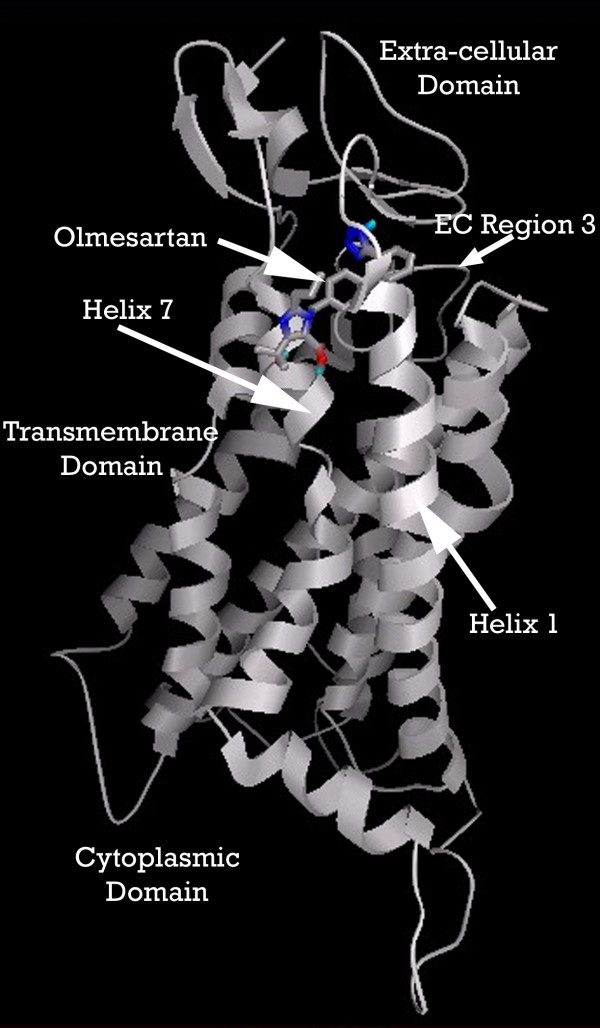
Overview of the ligand binding pocket identified in CCR2b (PDB:1KP1). Olmesartan is shown docked into pocket.

**Figure 14:**
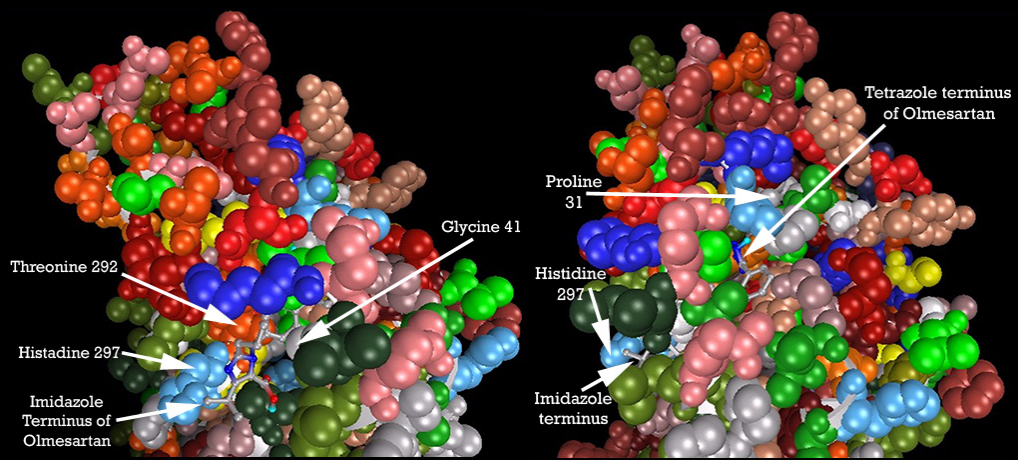
**Perspective view showing how pocket is located underneath Extracellular 'loop' 1. Olmesartan is shown docked into pocket**. Note: Residues displayed as 'CPK' charge spheres. Ligand displayed as stick and ball model. Left is view from front of pocket, facing helices 7 and 1, right view is from the top, looking across the top of helices 1 and 2.

Figure 13 shows the location of this binding pocket, and Figure 14 an overview of the pocket structure, running between GPCR helices seven and one, beneath the extracellular regionone, and bounded at the rear by extracellular region three.

**Figure 15:**
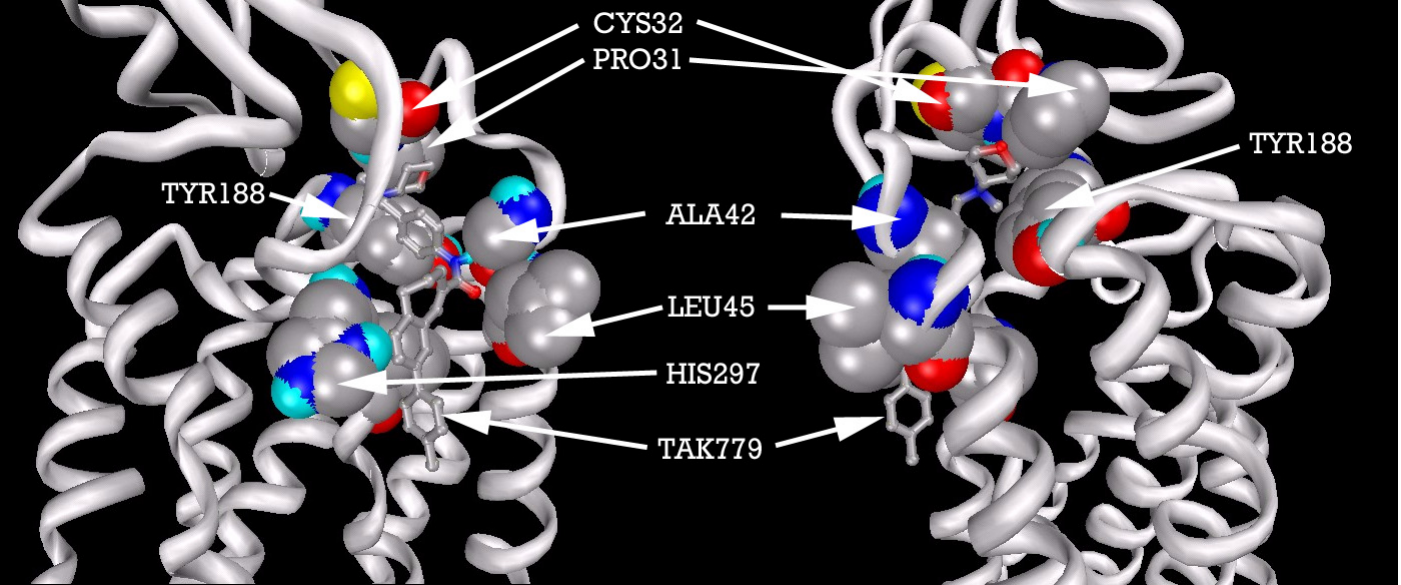
**CCR2b residues highlighted alongside docked TAK779. From left: front of pocket, rear of pocket**. Note: Carbon atoms shown as grey, oxygen as red, nitrogen as blue, polar hydrogen as blue-white, sulphur as yellow. Non-polar hydrogens not displayed. Residues displayed as 'CPK' charge spheres, ligand as 'ball and stick' models.

**Figure 16:**
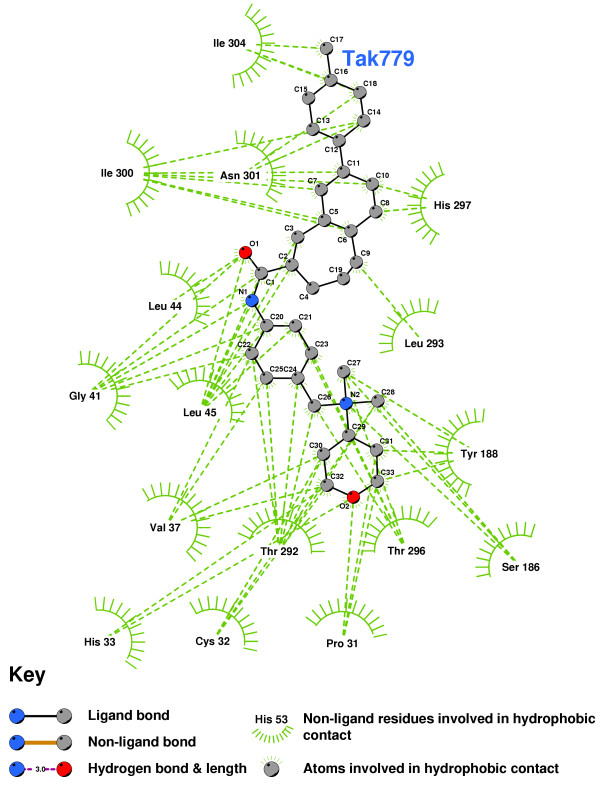
TAK779 docked into the CCR2b binding pocket.

Figure 15 shows the residues binding TAK779 into the putative pocket. Hydrophobic interactions with LEU45, HIS297, ILE300, TYR188, PRO31 and CYS32, help to stabilize the ligand. The 2D LigPlot of residue interactions can be seen at Figure 16.

Olmesartan and Irbesartan each showed excellent affinity (Ki≈9 nmol) for this binding pocket, while Valsartan, Telmisartan, Candesartan and Losartan exhibited slightly less (Kifrom 22 to 40 nmol).

**Figure 17:**
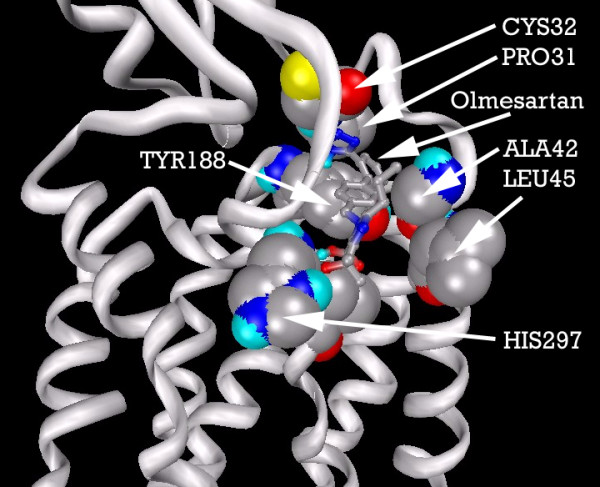
**CCR2b residues highlighted alongside docked Olmesartan, viewed from the front of the binding pocket**. Note: Carbon atoms shown as grey, oxygen as red, nitrogen as blue, polar hydrogen as blue-white, sulphur as yellow. Non-polar hydrogens not displayed. Residues displayed as 'CPK' charge spheres, ligand as 'ball and stick' models.

**Figure 18:**
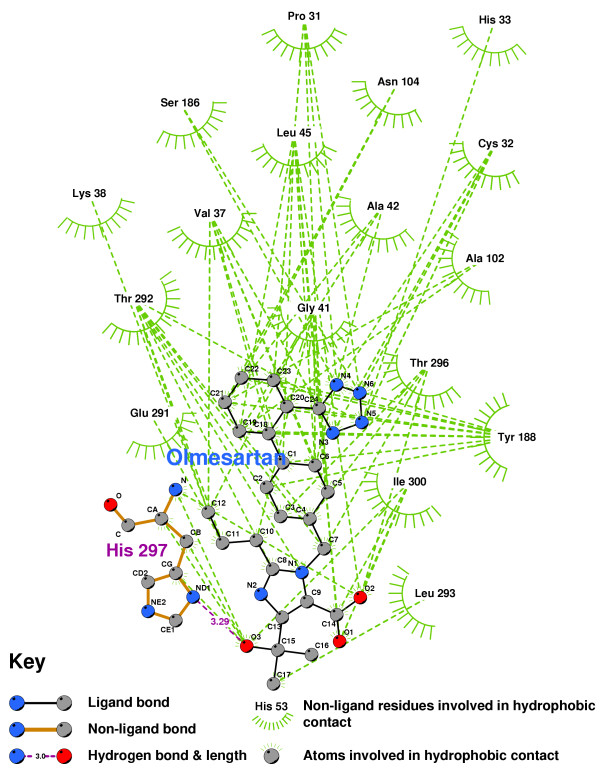
**Olmesartan docked into the CCR2b binding pocket**. Note: The core structure of the hydrogen-bonded residues is expanded to a 'ball-and-stick' format, so as to show the atoms involved in hydrogen bond formation.

Figure 17 shows the residues which interact with Olmesartan. A hydrogen bond is formed with the imidazole of HIS297, while ILE300, ALA42, LEU45, THR292, TYR188, CYS32 and PRO31 all help to stabilize the ligand. Figure 18 shows the 2D LigPlot of these interactions.

**Figure 19:**
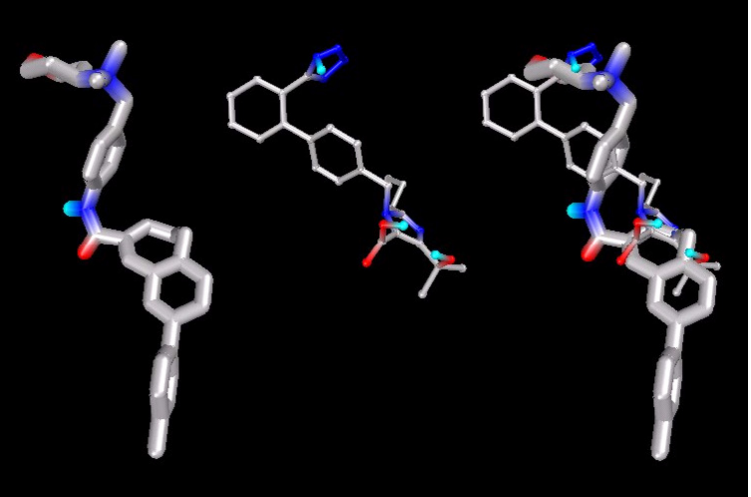
**CCR2b-docked configurations for TAK779 and Olmesartan, individually and with superimposition**. Note: Ligands depicted as "thick" and "thin" solely for visual clarity. Carbon atoms shown as grey, oxygen as red, nitrogen as blue, polar hydrogen as blue-white. Non-polar hydrogens not displayed.

Figure 19 shows the docked position of TAK779 and Olmesartan superimposed, to enable easier comparison of the final location of each ligand.

**Figure 20:**
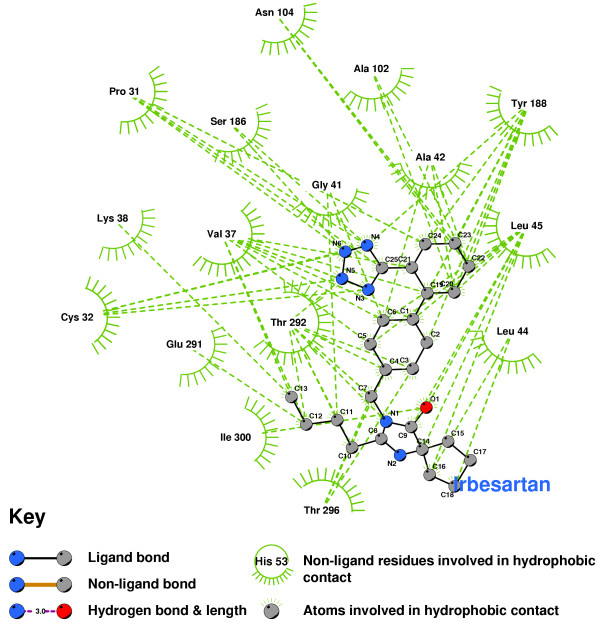
Irbesartan docked into the CCR2b binding pocket.

Irbesartan forms hydrophobic contacts with a set of residues similar to that of Olmesartan (see Figure 20).

The ARBs, and TAK779, not only fill space within this binding pocket, but also 'anchor' the top of helices seven and one to extracellular regions three and one, restraining the motion of GPCR elements, and, most probably, inhibiting its activation [43].

### A putative AT2R1 receptor model

A primary goal set for this study had been the validation of every structure and tool we used. It had therefore been decided to ensure that the ARBs would dock into AT2R1 with inhibition constants close to the values measured in-vitro, as documented in the various FDA New Drug Applications (NDAs). For example, NDA21-286 [2], indicates a Ki for Olmesartan and Candesartan of approx. 0.1 nanomolar, and for Losartan about 3 times higher.

This validation task proved to be the most difficult of the study. There was no AT2R1 X-ray structure publicly available, nor any comprehensive theoretical model. Additionally, there was very little comparative experimental ARB data available (FDA NDA21-286 is the exception to this). Most authors studied only one commercial ARB product in isolation.

We tried to use the theoretical model published by Martin, et.al. [43] [PDB:1ZV0] for an activated AT2R1. But no ARB would bind to that receptor configuration, even after the extensive energy optimization required to move helix seven back into its un-activated position.

We then decided to produce an AT2R1 model by comparative homology [40] with Bovine Rhodopsin [PDB:1L9H], but still could not produce a model which would dock the known ARBs, even after extensive energy minimization. Eventually we used the putative CCR2b, [PDB:1KP1] as the comparative model. Surprisingly, straight out of the 'Modeller' [41], all the ARBs docked into a pocket on the opposite side of the GPCR from the binding pocket which had been located on CCR2b. The Ki for the ARBs ranged from 0.10 to 1.5 nmol, as detailed in Table 1.

It is interesting to note that although the comparative homology between AT2R1 and Rhodopsin is only 17% (Table 2) the AT2R1 sequence is much closer to that of CCR2b (Table 4). Our failure to produce a usable receptor by comparative homology with Bovine Rhodopsin would seem to caste doubt on its utility as a prototype for the Class A 7-transmembrane GPCR structures.

**Figure 21:**
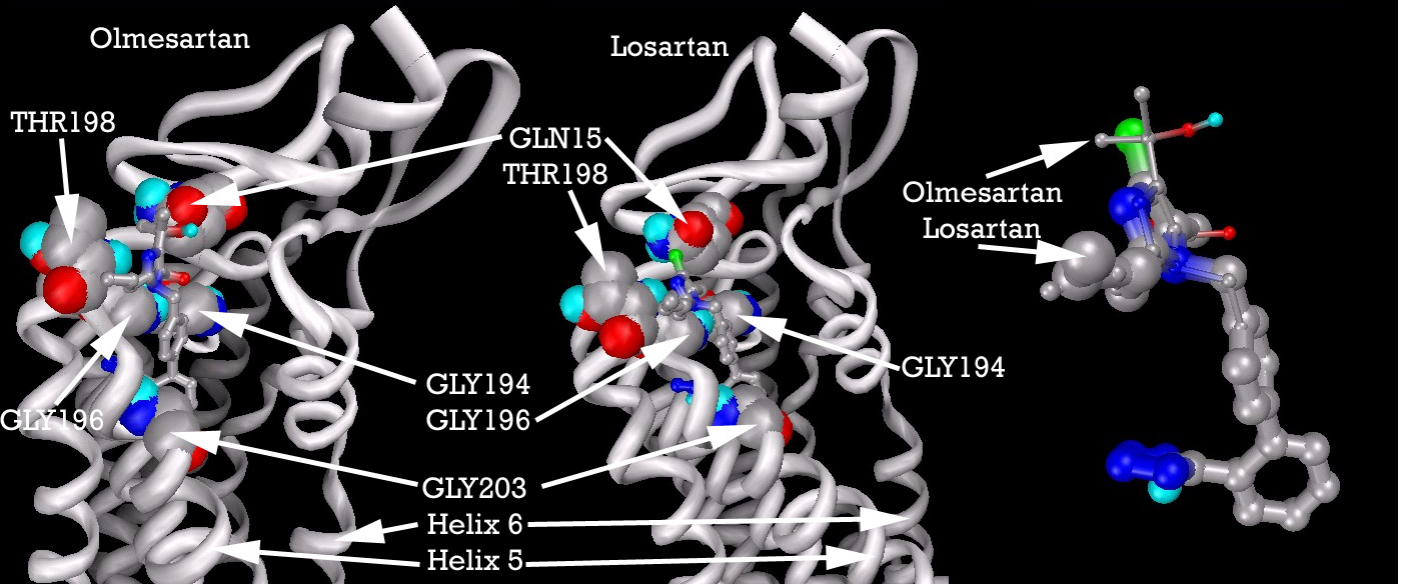
**Putative AT2R1 with (from left) Olmesartan, and Losartan docked, showing primary residues. Ligands are also shown superimposed**. Note: Carbon atoms shown as grey, oxygen as red, nitrogen as blue, polar hydrogen as blue-white, and chlorine as green. Non-polar hydrogens not displayed. Residues displayed as 'CPK' charge spheres, ligands as 'ball and stick' models. Thick and thin ligand backbones displayed solely for visual clarity.

Figure 21 shows the primary residues involved in docking the ARBs, and a superimposition of the docked conformations of Olmesartan and Losartan, demonstrating the homogeneity of location of the imidazole group into the binding pocket, even amongst ARBs with significant structural differences.

**Figure 22:**
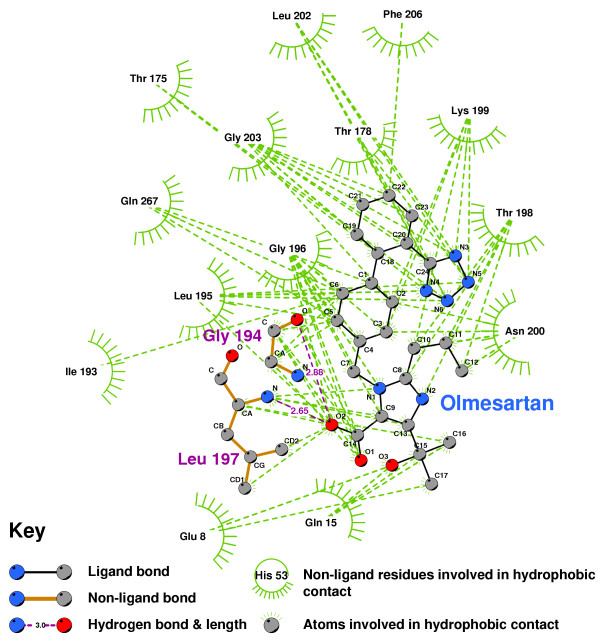
**Olmesartan docked into the putative AT2R1 binding pocket**. Note: The core structure of the hydrogen-bonded residues is expanded to a 'ball-and-stick' format, so as to show the atoms involved in hydrogen bond formation.

**Figure 23:**
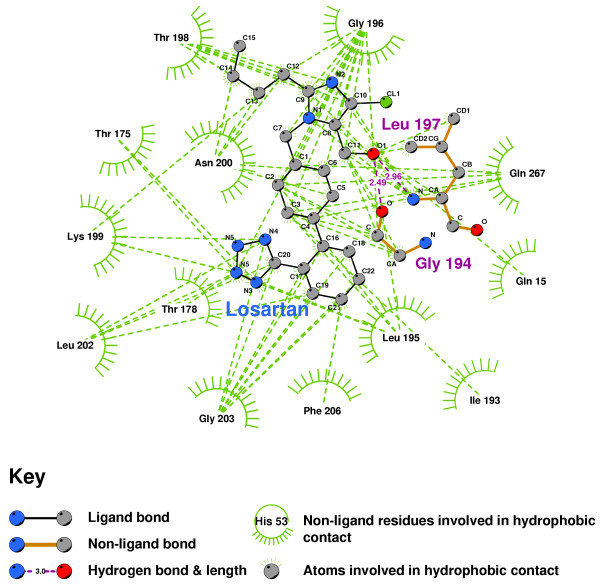
**Losartan docked into the putative AT2R1 binding pocket**. Note: The core structure of the hydrogen-bonded residues is expanded to a 'ball-and-stick' format, so as to show the atoms involved in hydrogen bond formation.

**Figure 24:**
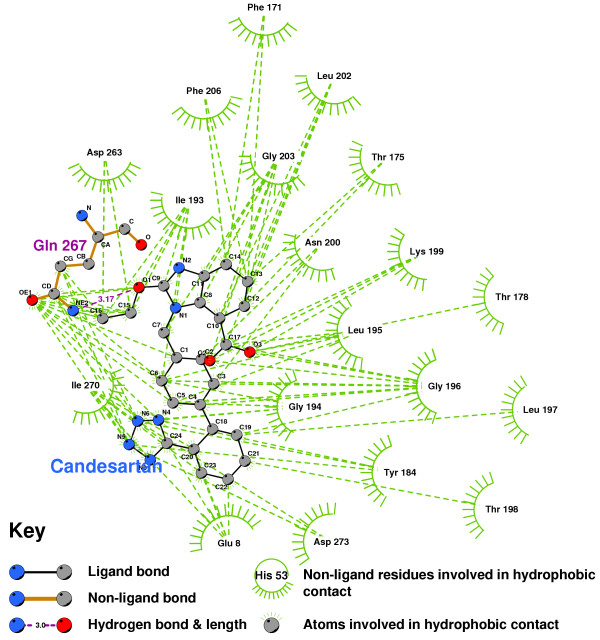
**Candesartan docked into the putative AT2R1 binding pocket**. Note: The core structure of the hydrogen-bonded residues is expanded to a 'ball-and-stick' format, so as to show the atoms involved in hydrogen bond formation.

The hydrophobic interactions between Olmesartan and our AT2R1 is shown in Figure 22. Olmesartan forms two hydrogen bonds, with GLY194 and LEU197, as does Losartan (Figure 23). Candesartan binds to quite different residues, in particular, making 6 hydrophobic contacts with ILE193(Figure 24).

## Discussion

### Models provided to ease visualization of nuclear receptors

It is evident from the lack of clarity in Figure 4 that it is extremely difficult to visualize ligand conformation in the binding pockets of nuclear receptors using two dimensional media. For this reason we have provided, as an attached file, an archive of the receptor configurations used in this study, in addition to the most significant bound ligand conformations. The models can be loaded into, for example, the Python Molecular Viewer [35], and 3D analysis performed.

This archive will also facilitate the testability of our results.

### Does telmisartan selectively modulate PPARgamma?

Benson, et.al. [20], presented the ARBs as suited to PPARgamma modulation. Their primary conclusion was that Telmisartan's structure allowed it to exhibit selective modulation, exhibiting in-vitro PPARgamma agonistic activity at low concentrations, changing to antagonistic activity at higher concentrations.

**Figure 25:**
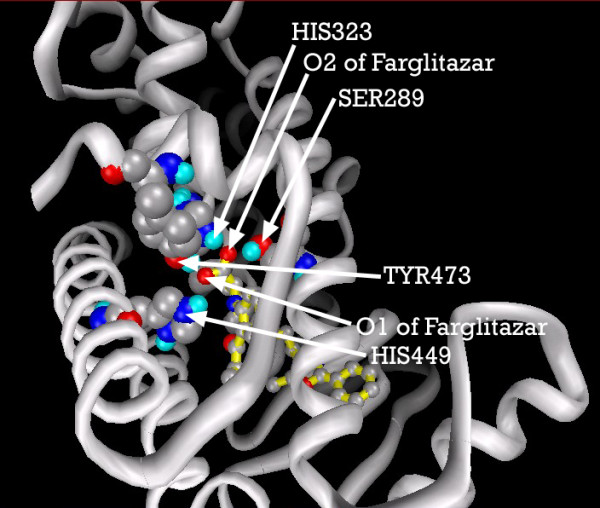
**Farglitazar docked into the PPARgamma ligand binding pocket, showing the primary residues involved in hydrogenbonding**. Note: Ligand depicted with yellow backbone solely for visual clarity. Carbon atoms shown as grey, oxygen as red, nitrogen as blue, polar hydrogen as blue-white. Non-polar hydrogens not displayed. Residues displayed as 'CPK' charge spheres, ligand as 'ball and stick' model.

**Figure 26:**
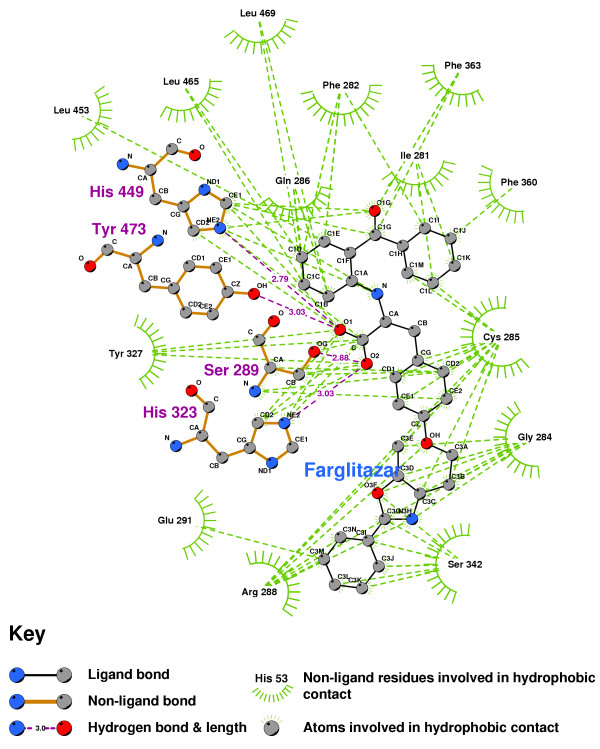
**Farglitazar docked into the PPARgamma ligand binding domain**. Note: The core structure of the hydrogen-bonded residues is expanded to a 'ball-and-stick' format, so as to show the atoms involved in hydrogen bond formation.

Figure 25 shows the key binding pocket for the agonist Farglitazar (GI262570) in the PPAR ligand binding domain. Figure 26, the LigPlot of this conformation, shows two key hydrogen bonds between Farglitazar's O1, HIS449 and TYR473, and two more between O2, SER289 and HIS323.

Tsukahara, et.al. [52] recently studied a number of PPAR agonists. They found that agonistic activity disappears when TYR473 is mutated, and noted the importance of HIS323 and HIS449.

**Figure 27:**
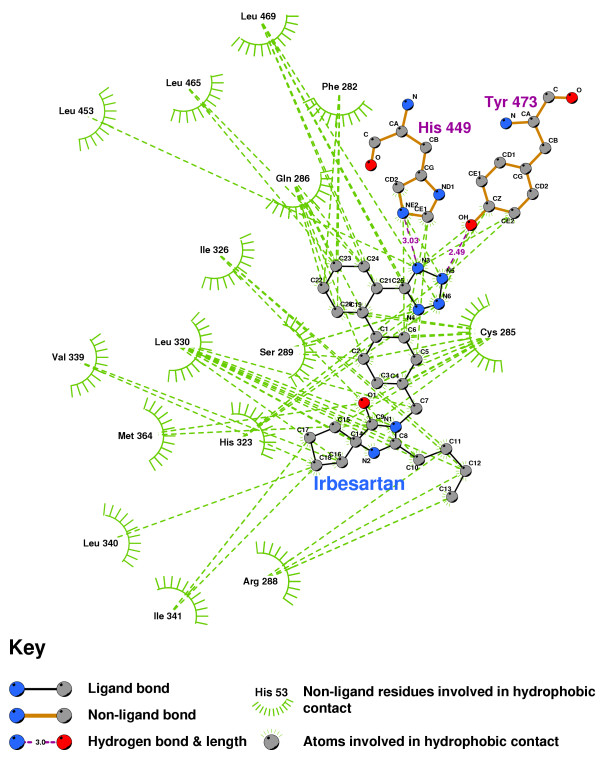
**Irbesartan docked into the PPARgamma ligand binding domain**. Note: The core structure of the hydrogen-bonded residues is expanded to a 'ball-and-stick' format, so as to show the atoms involved in hydrogen bond formation.

**Figure 28:**
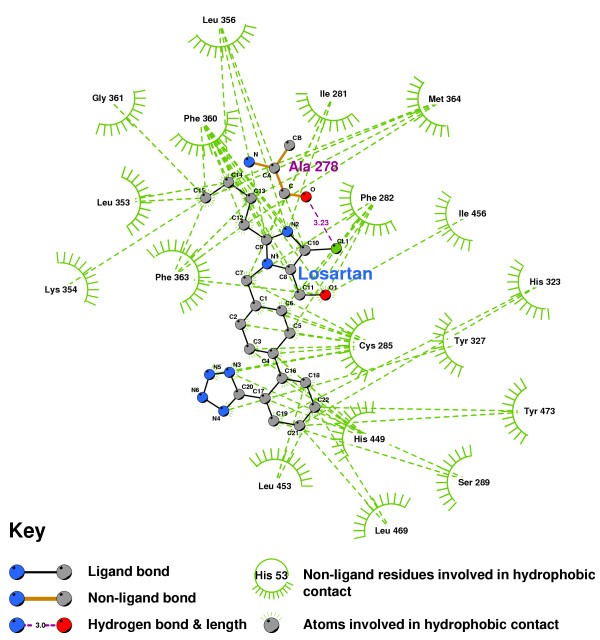
**Losartan docked into the PPARgamma ligand binding domain**. Note: The core structure of the hydrogen-bonded residues is expanded to a 'ball-and-stick' format, so as to show the atoms involved in hydrogen bond formation.

Figures 27 and 28 show the residues which contact PPARgamma when Irbesartan and Losartan are docked into their minimum energy conformations. Although Irbesartan hydrogen-bonds TYR473 and HIS449, Losartan only contacts these residues, and forms its sole hydrogen-bond to ALA278. It would thus seem likely that Losartan is an effective PPAR antagonist. Irbesartan does not hydrogen-bond to HIS323, a residue found critical to Rosiglitazar's agonism [52], and probably is more likely an antagonist than agonist.

**Figure 29:**
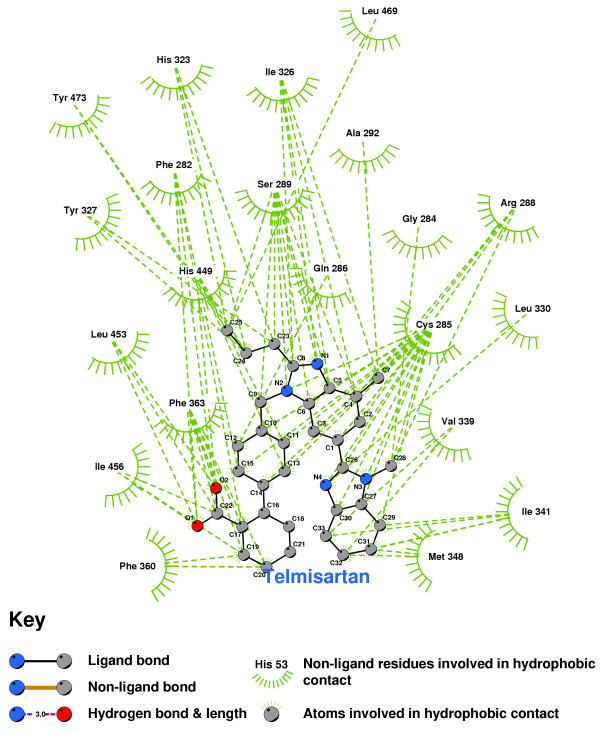
Telmisartan docked into the PPARgamma ligand binding domain.

Figure 29 shows that Telmisartan does not form any hydrogen bonds with the PPARgamma residues identified by Tsukahara, et.al., as critical to the agonistic activity of Rosglitazar. Any molecular mechanism which could result in 'partial agonism' of PPARgamma by Telmisartan is still to be elucidated

We would note, however, that the extreme affinity which Telmisartan exhibits for the ubiquitous VDR might well alter expression of many hormones at concentrations lower than those at which Telmisartan begins to modulate PPARgamma. This may make it very difficult to evaluate cause and effect in the cascade of metabolic changes which will result from Telmisartan's blockade of the VDR.

### Bovine and guinea pig AT2R1 for FDA in-vitro ARB studies

While modelling the ARBs docking into the AT2R1 receptor, we were struck by data in United States Food and Drug Administration (US FDA) documents which did not exactly match our own observations.

For example, there are inconsistencies between our predictions for the relative efficacies of Olmesartan, Candesartan and Losartan; and those of Figure 1.1.1.4 of FDA NDA21-286 [2]. The NDA's in-vitro experiments, using *Cavia porcellus*, showed Olmesartan as having the highest ARB efficacy, as we did, but found Candesartan close in efficacy to Olmesartan (1.2×) and Losartan to be less effective (3.4×). Our study found Losartan (Ki≈0.5 nmol) to be a better antagonist of AT2R1 than was Candesartan (Ki≈1.5 nmol).

The answer may well lie in sequence divergence between the AT2R1 proteins from human, bovine, and guinea pig sources. The multiple sequence alignment showing differences between AT2R1 from *Homo sapiens*, *Cavia porcellus *and *Bos taurus *is shown in Table 5.

**Table 5:** Multiple sequence alignment highlighting differences between AT2R1 from *Homo sapiens*, *Cavia porcellus *and *Bos taurus*.

sp|P30556|AGTR1_HUMAN gi|8927995|sp|Q9WV26|AGTR1_CAV gi|27806329|ref|NP_776658.1|B.taurus	
sp|P30556|AGTR1_HUMAN gi|8927995|sp|Q9WV26|AGTR1_CAV gi|27806329|ref|NP_776658.1|B.taurus	
sp|P30556|AGTR1_HUMAN gi|8927995|sp|Q9WV26|AGTR1_CAV gi|27806329|ref|NP_776658.1|B.taurus	
sp|P30556|AGTR1_HUMAN gi|8927995|sp|Q9WV26|AGTR1_CAV gi|27806329|ref|NP_776658.1|B.taurus	
sp|P30556|AGTR1_HUMAN gi|8927995|sp|Q9WV26|AGTR1_CAV gi|27806329|ref|NP_776658.1|B.taurus	
sp|P30556|AGTR1_HUMAN gi|8927995|sp|Q9WV26|AGTR1_CAV gi|27806329|ref|NP_776658.1|B.taurus	
sp|P30556|AGTR1_HUMAN gi|8927995|sp|Q9WV26|AGTR1_CAV gi|27806329|ref|NP_776658.1|B.taurus	
sp|P30556|AGTR1_HUMAN gi|8927995|sp|Q9WV26|AGTR1_CAV gi|27806329|ref|NP_776658.1|B.taurus	

Our model predicts that the primary residues involved in docking most of the ARBs are GLN15, GLY194, GLY196, THR198 and GLY203.

The binding pocket around GLN15 is conserved in all three homologies.

However, in *Bos taurus*, the Isoleucine residue 193 is mutated to Valine. Candesartan has 6 hydrophobic contacts with ILE193, while Losartan and Olmesartan have only one. It is thus very likely that substitution of ILE193 will differentially effect the degree of Candesartan's antagonism of *Bos taurus *AT2R1 receptors, when compared with that of other ARBs, less dependent on contacts with ILE193.

Additionally, there is a mutation in Leucine 205, structurally adjacent to GLY203. GLY203 has seven hydrophobic contacts with Olmesartan, eight with Losartan, and six with Candesartan. In *Cavia porcellus*, this Glycine is mutated to Methionine.

The authors consequently believe that the FDA should re-examine the acceptability of *Bos taurus *and *Caviaporcellus *tissues for demonstration of the efficacy of ARBs.

It was beyond the scope of this study to model AT2R1 receptors for all three species used in the FDA in-vitro data. This should form a topic for ongoing research.

## Conclusion

The FDA-approved prescribing information for Valsartan states "Valsartan does not bind to or block other hormone receptors or ion channels known to be important in cardiovascular regulation." This is an accurate statement of current knowledge about ARB in-vivo activity.

Yet this study found Valsartan (and the other ARBs) had a profound affinity for the hormone receptor VDR, for PPARgamma and for CCR2b. Clearly, if our modelling data sustains validation in the laboratory, clinical medicine will need to re-examine current concepts of how ARBs function in-vivo. It is possible that ARBs may become useful as potent immunomodulatory agents in addition to their current indication as cardiovascular drugs. This study has shown how each ARB acts upon several key receptors of the immune system, and should serve as a solid basis for better understanding the anti-inflammatory properties of this class of pharmaceutical.

## Methods

### Hardware and molecular tools

Two network servers were configured with Debian Linux. 'AutoDock' [25,26,43,44] was kindly supplied by Scripps' and 'Modeller' by Salilab [41]. Ponder's 'Tinker' Toolset was downloaded from the cited location [39]. The Fortran software sources for 'Modeller' and 'Tinker' were recompiled with 64-bit Athlon-class optimizations applied, to suit the 64-bit CPU. Each server had 1 Gigabyte of memory, and a 160 Gigabyte hard disk. They were networked (using Samba [50]) to the primary Windows 2000 based workstation. The workstation also ran AutoDock (using the Cygwin executables), Python Molecular Viewer [35], and AutoDock Tools [34].

### Optimization of the modelling software parameters

Autodock uses a default grid size of 0.375 Angstroms. This was changed to 0.2 Angstroms, noticeably improving upon the Ki calculated with the coarser grid. However, the computing time with this more precise grid was increased four-fold. To ensure more reliable minima from AutoDock's Lamarckian genetic algorithm, the 'population size' parameter "ga_pop_size" was increased from 50 to 100, and the number of energy calculations for each set, "ga_num_evals," was increased from 250,000 to 1,000,000. One set of AutoDock grid maps was typically generated for each receptor, and multiple ligands were docked without changing the grid maps. Docking parameter files were edited using the Linux ASCII text editor.

Energy minimization of structures with Ponder's 'minimize' and 'pss' [38] programs was effected using the 'Amber99' parameter set [47].

### Construction of ligand and receptor molecules

Akira Dobashi's 3D Pharmaceutical Structure Database at Pharmis.org [48] (Tokyo University of Pharmacy and Life Sciences) was the primary source of ARB models. Olmesartan had to be built with Ghemical [49], running on a Linux server. Receptor coordinates were taken from the RCSB Protein Databank (PDB), or generated using Modeller [41] (as detailed in the text).

### LigPlot and HBPLUS

McDonald's HBPLUS software [56,55] takes, as input, the computed 3D ligand-receptor complex and produces a table of hydrogen bonds formed between the ligand and the receptor. It also produces tables of non-bonding hydrophobic contacts between the ligand atoms and receptor residues (default distance parameters were used for both bonds and contacts). Wallace and Laskowski's LigPlot software [53,54] takes those tables and creates a 2D representation of the bonds and contacts, iteratively optimizing the output against a set of user-specified plot parameters. For example, weight-parameters can be assigned to minimize areas where the plot of hydrophobic bonds becomes too dense, forcing LigPlot to iteratively move the 2D positions of the residues so as to minimize that clutter, and thus make the output more readable. The output of Ligplot is PostScript, which was modified with a text editor to maximize font readability, and crop excess white space.

## Competing interests

REL has no competing interests. TGM is designated as inventor on a US patent application titled "Treatment of Th1 and autoimmune diseases effected with angiotensin inhibition and antibiotics." No assistance has been requested or received by any of the authors from any pharmaceutical company, or other financially interested entity. This study was entirely funded by the authors.

## Authors' contributions

TGM conceived, designed, and carried out the molecular studies, performed the 'Modeller' sequence alignments, configured the computer servers, the computer software, and drafted the manuscript. REL was responsible for receptor phylogenies and Clustal alignments. FEM participated in the molecular model definition, coordinated the FDA and pharmacological issues, and helped to draft the manuscript. All authors read and approved the final manuscript.

## Supplementary Material

Additional File 1 **'ARB-immune-models.tar.gz' – Models of receptors and significant ligands**. This is a Tar-Gzip archive which can by unpacked by using 'tar-xvzf', Winzipv8+ or 'Mac Stuffit'. There are five directories within it, containing a total of 35 files, 1.54 Meg when unpacked, 360 K when compressed: • 'AT2R1' contains the receptor model described in this paper, plus the docked conformation of each ARB, corresponding to the Ki values in Table 1. • 'CCR2b' contains the receptor model we derived from 1KP1, together with TAK779 and each of the ARBs in their docked conformation. • 'PPAR' contains the receptor model we derived from PDB:1FM9 together with the GI262570 ligand from PDB:1FM9 and each of the ARBs which have low Ki values when docked with the receptor. • 'VDR_from_1DB1" contains the VDR model we derived from PDB:1DB1 along with the docked conformations of the ARBs, 1,25-D (from PDB:1DB1) and TX522 (from PDB:1TXI). • 'VDR_from_1TXI' contains the VDR model we derived from PDB:1TXI along with the docked conformation of TX522, 1,25-D and Telmisartan. Click here for file

## References

[B1] Mann DL, Deswal A Angiotensin-receptor blockade in acute myocardial infarction – a matter of dose. N Engl J Med.

[B2] United States Food and Drug Administration Approval Package for NDA21-286. http://www.fda.gov/cder/foi/nda/2002/21-286_Benicar_pharmr_P1.pdf.

[B3] Izuhara Y, Nangaku M, Inagi R, Tominaga N, Aizawa T, Kurokawa K, van Ypersele de Strihou C, Miyata T (2005). Renoprotective Properties of Angiotensin Receptor Blockers beyond Blood Pressure Lowering. J Am Soc Nephrol.

[B4] Lewis EJ, Lewis JB (2003). Treatment of diabetic nephropathy with angiotensin II receptor antagonist. Clin Exp Nephrol.

[B5] Brenner BM, Cooper ME, de Zeeuw D, Keane WF, Mitch WE, Parving HH (2001). Effects of losartan on renal and cardiovascular outcomes in patients with type 2 diabetes and nephropathy. N Engl J Med.

[B6] Viberti GC, Wheeldon MN (2002). Microalbuminuria reduction with valsartan in patients with type 2 diabetes mellitus: A blood pressure-independent effect. Circulation.

[B7] Schieffer B, Bunte C, Witte J, Hoeper K, Boger RH, Schwedhelm E, Drexler H Comparative effects of AT1-antagonism and angiotensin-converting enzyme inhibition on markers of inflammation and platelet aggregation in patients with coronary artery disease. J Am Coll Cardiol.

[B8] Luno J, Praga M, de Vinuesa SG (2005). The reno-protective effect of the dual blockade of the renin angiotensin system (RAS). Curr Pharm Des.

[B9] Waterhouse JC, Marshall TG, Fenter B, Mangin M, Blaney G, Stoltz VD (2006). High levels of active 1,25-dihydroxyvitamin D despite low levels of the 25-hydroxyvitamin D precursor - Implications of dysregulated vitamin D for disgnosis and treatment of Chronic Disease. Vitamin D: New Research.

[B10] Marshall TG, Marshall FE (2004). Sarcoidosis succumbs to antibiotics – implications for autoimmune disease. Autoimmun Rev.

[B11] Raz E, Tighe H, Sato Y, Corr M, Dudler JA, Roman M, Swain SL, Spiegelberg HL, Carson DA Preferential induction of a Th1 immune response and inhibition of specific IgE antibody formation by plasmid DNA immunization. Proc Natl Acad Sci USA.

[B12] Cantorna MT, Zhu Y, Froicu M, Wittke A (2004). Vitamin D status, 1,25-dihydroxyvitamin D3, and the immune system. Am J Clin Nutr.

[B13] Mallat Z, Ait-Oufella H, Tedgui A (2005). Regulatory T cell responses: potential role in the control of atherosclerosis. Curr Opin Lipidol.

[B14] Yoon JW, Jun HS (2005). Autoimmune destruction of pancreatic Beta cells. Am J Ther.

[B15] Wittke A, Weaver V, Mahon BD, August A, Cantorna MT Vitamin D receptor-deficient mice fail to develop experimental allergic asthma. J Immunol.

[B16] Dusso AS, Kamimura S, Gallieni M, Zhong M, Negrea L, Shapiro S, Slatopolsky E (1997). gamma-Interferon-induced resistance to 1,25-(OH)2 D3 in human monocytes and macrophages: a mechanism for the hypercalcemia of various granulomatoses. J Clin Endocrinol Metab.

[B17] Potashnik G, Lunenfeld E, Levitas E, Itskovitz J, Albutiano S, Yankowitz N, Sonin Y, Levy J, Glezerman M, Shany S (1992). The relationship between endogenous oestradiol and vitamin D3 metabolites in serum and follicular fluid during ovarian stimulation for in-vitro fertilization and embryo transfer. Hum Reprod.

[B18] Chatterjee M Vitamin D and genomic stability. Mutat Res.

[B19] Nagpal S, Na S, Rathnachalam R (2005). Noncalcemic actions of vitamin D receptor ligands. Endocr Rev.

[B20] Benson SC, Pershadsingh HA, Ho CI, Chittiboyina A, Desai P, Pravenec M, Qi N, Wang J, Avery MA, Kurtz TW (2004). Identification of telmisartan as a unique angiotensin II receptor antagonist with selective PPARgamma-modulating activity. Hypertension.

[B21] Cabrero A, Laguna JC, Vazquez M (2002). Peroxisome proliferator-activated receptors and the control of inflammation. Curr Drug Targets Inflamm Allergy.

[B22] Genolet R, Wahli W, Michalik L (2004). PPARs as drug targets to modulate inflammatory responses?. Curr Drug Targets Inflamm Allergy.

[B23] Preobrazhensky AA, Dragan S, Kawano T, Gavrilin MA, Gulina IV, Chakravarty L, Kolattukudy PE Monocyte chemotactic protein-1 receptor CCR2B is a glycoprotein that has tyrosine sulfation in a conserved extracellular N-terminal region. J Immunol.

[B24] Tanaka S, Green SR, Quehenberger O Differential expression of the isoforms for the monocyte chemoattractant protein-1 receptor, CCR2, in monocytes. Biochem Biophys Res Commun.

[B25] Casey PJ, Gilman AG G protein involvement in receptor-effector coupling. J Biol Chem.

[B26] AutoDock, Automatic Docking of Flexible Ligands to Macromolecules. http://www.scripps.edu/mb/olson/doc/autodock/.

[B27] Morris GM, Goodsell DS, Halliday RS, Huey R, Hart WE, Belew RK, Olson AJ Automated docking using Lamarckian genetic algorithm and an empirical binding free energy function. J Comp Chem.

[B28] Osterberg F, Morris GM, Sanner MF, Olson AJ, Goodsell DS Automated docking to multiple target structures: incorporation of protein mobility and structural water heterogeneity in AutoDock. Proteins.

[B29] Toprakci M, Yelekci K Docking studies on monoamine oxidase-B inhibitors: estimation of inhibition constants (K(i)) of a series of experimentally tested compounds. Bioorg Med Chem Lett.

[B30] Chen K, Adelstein SJ, Kassis AI (2004). Molecular simulation of ligand-binding with DNA: implications for 125I-labeled pharmaceutical design. Int J Radiat Biol.

[B31] Xu HE, Lambert MH, Montana VG, Plunket KD, Moore LB, Collins JL, Oplinger JA, Kliewer SA, Gampe RT, McKee DD, Moore JT, Willson TM Structural determinants of ligand binding selectivity between the peroxisome proliferator-activated receptors. Proc Natl Acad Sci U S A.

[B32] Rochel N, Wurtz JM, Mitschler A, Klaholz B, Moras D (2000). The crystal structure of the nuclear receptor for vitamin D bound to its natural ligand. Mol Cell.

[B33] Eelen G, Verlinden L, Rochel N, Claessens F, De Clercq P, Vandewalle M, Tocchini-Valentini G, Moras D, Bouillon R, Verstuyf A (2005). Superagonistic action of 14-epi-analogs of 1,25-dihydroxyvitamin D explained by vitamin D receptor-coactivator interaction. Mol Pharmacol.

[B34] Scripps Research Institute, Molecular Graphics Laboratory, MGLtools. http://www.scripps.edu/~sanner/software/.

[B35] The Python Molecular Viewer. http://www.pymol.org.

[B36] Strader CD, Fong TM, Graziano MP, Tota MR (1995). The family of G-protein-coupled receptors. FASEB J.

[B37] Shi XF, Liu S, Xiangyu J, Zhang Y, Huang J, Liu S, Liu CQ (2002). Structural analysis of human CCR2b and primate CCR2b by molecular modeling and molecular dynamics simulation. J Mol Model (Online).

[B38] Pappu RV, Marshall GR, Ponder JW (1999). A potential smoothing algorithm accurately predicts transmembrane helix packing. Nat Struct Biol.

[B39] TINKER – Software tools for molecular design. http://dasher.wustl.edu/tinker.

[B40] Sali A, Blundell TL Comparative protein modelling by satisfaction of spatial restraints. J Mol Biol.

[B41] MODELLER Program for comparative protein structure modelling by satisfaction of spatial restraints. http://salilab.org/modeller/modeller.html.

[B42] Baba M, Nishimura O, Kanzaki N, Okamoto M, Sawada H, Iizawa Y, Shiraishi M, Aramaki Y, Okonogi K, Ogawa Y, Meguro K, Fujino M A small-molecule, nonpeptide CCR5 antagonist with highly potent and selective anti-HIV-1 activity. Proc Natl Acad Sci USA.

[B43] Martin SS, Boucard AA, Clement M, Escher E, Leduc R, Guillemette G Analysis of the third transmembrane domain of the human type 1 angiotensin II receptor by cysteine scanning mutagenesis. J Biol Chem.

[B44] Goodsell DS, Olson AJ (1990). Automated Docking of Substrates to Proteins by Simulated Annealing. Proteins.

[B45] Morris GM, Goodsell DS, Huey R, Olson AJ (1996). Distributed Automated Docking of Flexible Ligands to Proteins: Parallel Applications of AutoDock 2.4. J Comput Aided Mol Des.

[B46] Omdahl JL, Morris HA, May BK (2002). Hydroxylase enzymes of the vitamin D pathway: expression, function, and regulation. Annu Rev Nutr.

[B47] Wang J, Cieplak P, Kollman PA (2000). How Well Does a Restrained Electrostatic Potential (RESP) Model Perform in Calcluating Conformational Energies of Organic and Biological Molecules?. J Comput Chem.

[B48] Akira Dobashi's 3D Pharmaceutical Structure Database.

[B49] Ghemical molecular modelling package. http://www.bioinformatics.org/ghemical/.

[B50] Blair JD (1998). SAMBA: Integrating UNIX and Windows.

[B51] Acevedo A, Stoynova L, Davis K, Solorzano R, Collins ED (2004). Role of residues 143 and 278 of the human nuclear Vitamin D receptor in the full-length and Delta165-215 deletion mutant. J Steroid Biochem Mol Biol.

[B52] Tsukahara T, Tsukahara R, Yasuda S, Makarova N, Valentine WJ, Allison P, Yuan H, Baker DL, Li Z, Bittman R, Parrill A, Tigyi G Different residues mediate recognition of 1-O-oleyl-lysophosphatidic acid and rosiglitazone in the ligand binding domain of PPAR1. J Biol Chem.

[B53] Wallace AC, Laskowski RA, Thornton JM (1995). A program to generate schematic diagrams of protein-ligand interactions. Protein Eng.

[B54] LIGPLOT-Program for automatically plotting protein-ligand interactions. http://www.biochem.ucl.ac.uk/bsm/ligplot/ligplot.html.

[B55] McDonald IK, Thornton JM Satisfying Hydrogen Bonding Potential in Proteins. J Mol Biol.

[B56] HBPLUS-Hydrogen Bond Calculation Program. http://www.biochem.ucl.ac.uk/bsm/hbplus/home.html.

[B57] Shaffer PL, McDonnell DP, Gewirth DT Characterization of transcriptional activation and DNA-binding functions in the hinge region of the vitamin D receptor. Biochemistry.

